# Head movement differs for positive and negative emotions in video recordings of sitting individuals

**DOI:** 10.1038/s41598-021-86841-8

**Published:** 2021-04-01

**Authors:** Maciej Behnke, Nadia Bianchi-Berthouze, Lukasz D. Kaczmarek

**Affiliations:** 1 Faculty of Psychology and Cognitive Science, Adam Mickiewicz University, 61-664 Poznan, Poland; 2 University College London, Interaction Centre, London, WC1E 6EA UK

**Keywords:** Psychology, Human behaviour

## Abstract

Individuals tend to approach positive stimuli and avoid negative stimuli. Furthermore, emotions influence whether individuals freeze or move more. These two kinds of motivated behavior refer to the approach/avoidance behavior and behavioral freezing/activation. Previous studies examined (e.g., using forced platforms) whether individuals' behavior depends on stimulus' valence; however, the results were mixed. Thus, we aimed to test whether emotions' effects on spontaneous whole-body behavior of standing individuals also occur in the seated position. We used a computer vision method to measure the head sway in video recordings that offers ease of use, replicability, and unobtrusiveness for the seated research participant. We analyzed behavior recorded in the laboratory during emotion manipulations across five studies totaling 932 participants. We observed that individuals leaned more forward and moved more when watching positive stimuli than when watching negative stimuli. However, individuals did not behave differently when watching positive or negative stimuli than in the neutral condition. Our results indicate that head movements extracted from seated individuals' video recordings can be useful in detecting robust differences in emotional behavior (positive vs. negative emotions).

## Introduction

Individuals tend to lean and move towards positively valenced stimuli (approach behavior)^1,2^, and lean and move away from negatively valenced stimuli (avoidance behavior)^3–7^. Studying body sway has contributed to several applied contexts. For instance, body sway in response to affective stimuli was used to study posttraumatic stress^6^, aggression^8^, social threat^9^, and Parkinson's disease^10^. Most previous studies focused on standing participants. However, relatively less is known whether behavior of seated participants reveals the same emotional responses. Thus, our aim was to examine whether effects of valence on body sway also show up in head movement among seated participants. We focused on leaning forward vs. away and freezing vs. activation, dependent on whether individuals face positive (leaning forward and activation) or negative (leaning backward and freeze) stimuli. Moreover, whereas most previous studies used force-platforms^3,4,11,12^, we focused on video recordings to capitalize on more recent data analysis methods.

Body sway is a component of emotional behavior and is organized by its direction and intensity^13^. For direction, the biphasic approach to emotions suggests that emotion is organized around two motivational systems: approach oriented or appetitive and avoidance oriented or defensive^13^. For instance, individuals display avoidance behavior to pictures of mutilated bodies^3^, dangerous animals^4^, and guns^1^. In contrast, individuals display approach behavior to pictures of smiling individuals^2^, attractive individuals, beautiful landscapes, and delicious food^1^. Furthermore, for emotional behavior intensity, individuals decrease their body mobility facing negatively evaluated stimuli—response operationalized as behavioral freezing^14,15^. Freezing in response to aversive stimuli results from the adaptation to avoid threats^16,17^. Individuals reduce body movement when facing unpleasant stimuli, including social threat^9^, mutilation images^11,16^, anticipating electrical shock^18^, standing at height^19^, and recalling sad memories^20^. In contrast, individuals facing pleasant stimuli move more^21–24^. Activation in response to positive stimuli reflects the readiness to explore, play, or loosen up and decrease tension^5,25^. For instance, individuals activate and increase body movement when facing erotic scenes^21,22,24^, amusing traffic situations^23^, or watching pictures of offspring^22^.

Although most studies support the difference between negative and positive emotions, some studies did not find a differential influence of positive and negative emotions on behavior. For instance, some studies revealed no differences between positive and neutral conditions^4,9,12,24^ and between negative and neutral conditions^3,11,24^ in their influence on approach/avoidance behavior. However, in some, there was a significant difference between positive vs. negative emotions^9,24^. Moreover, for behavioral freezing/activation, some research indicated no differences between positive and neutral conditions^11,12,24^, between negative and neutral conditions^3,6,11,24^, and between negative and positive conditions^11,24^. These mixed findings indicate that the relationship between emotional behavior and valence is complex and easier to detect between extreme values (positive vs negative) than between extreme and middle values (e.g., positive vs. neutral)^23,24,26^.

The majority of research on body sway tested standing individuals (including unipedal stance) where shifts in center of pressure are most pronounced in the anterior–posterior direction during bipedal stance^9^ or during unipedal stance^11^. The research on body sway in a seated position is sparse^6,27^. Advancing methods of testing body sway in sitting individuals is important because individuals spend increasingly more time sitting, e.g., at work and leisure time^28^. Moreover, an increasing number of research focuses on new forms of activities that individuals perform while sitting (e.g., esports performance^29^). Thus, testing whether emotions' effects on standing individuals' behavior also occur in the more restricted seated position increases ecological validity and evidence diversity in the body sway research.

Furthermore, we focused on head movements as a commonly available movement index that can be extracted from video recordings. The head position relative to a stimulus is considered a critical output of seated postural responses^6^. Video recordings allow assessing the amount of spontaneous head and body sway of standing individuals with a reliability comparable to stabilometric force platform^24^. The advantage of using data extracted from video recordings comes from its low-cost and ease of use. Video data can be easily collected in laboratory experiments or ambulatory settings, including real-life scenarios.

In the present study, we reanalyzed five data sets totaling 931 participants (Table 1). We used video recordings from previous studies that were focused on the functions of positive emotions and cardiovascular responses^30–33^. We focused on dimensional conceptualizations of emotion (positive/negative) to address research investigating emotional experiences and their effects upon motor activity^34^. Thus, we focused on responses to positive, negative, and neutral stimuli. Moreover, we focused on two aspects of spontaneous emotional behaviors: direction (approach and avoidance behavior) and intensity (behavioral freezing/activation). The approach behavior is indicated by leaning forward and towards the stimulus, whereas avoidance behavior as moving away and reclining backward^35^. Behavioral freezing is defined as the reduction of body movement, whereas behavioral activation as an increase of body movement. Of note, these two features of behavior, i.e., direction and intensity, are relatively independent from each other. For instance, individuals facing a threat could recline backward and reduce their movement (avoidance and freezing behavior). In response to a neutral stimulus, individuals might neither recline nor lean forward but perform spontaneous nondirectional movements. Finally, to the positive stimuli, individuals might lean forward and perform more nondirectional movements. Building upon the previous research^1,9,21,22,24^, we expected that negative stimuli would influence individuals to move backward and move less (avoidance and freezing), whereas positive stimuli would influence individuals to lean forward and move more (approach and activation).

**Table 1:** Overview of studies characteristics.

Study number	Sample characteristics			Study characteristics			
	Size (% female)	Mean age (SD)	Participant pool (ethnicity)	Study procedure	Method	Originally elicited emotions	Design
Study 1	149 (53)	21.40 (2.67)	Undergraduates (Caucasian)	Baseline (5 min), series of six videos (all positive, all negative, or all neutral) (12 min), ultimatum game	Film clips	PE, NE, and NC	Between
Study 2	214 (49)	22.42 (2.68)	Romantic couples (Caucasian)	Baseline (5 min), three rounds of emotional film clips, and responses to partner success (capitalization)	Film clips	PE, NE, and NC	Between
Study 3	146 (70)	21.91 (2.45)	Undergraduates (Caucasian)	Baseline (5 min), speech preparation (30 s), pictures presentation (3 min), recovery	Pictures	High-approach PE, low-approach PE, and NC	Between
Study 4	189 (45)	21.83 (2.32)	Undergraduates (Caucasian)	Baseline (5 min), pictures presentation (3 min), speech preparation (3 min), recovery	Pictures	High-approach PE, low-approach PE, and NC	Between
Study 5	233 (0)	23.69 (3.53)	Gamers (Caucasian)	Baseline (5 min), five rounds of emotional film clip (random order; 2 min), video-game match (8 min), recovery (2 min)	Film clips	PE (amusement, enthusiasm), NE (anger, sadness), and NC	Within

*PE* positive emotions, *NE* negative emotions, *NC* neutral condition.

## Results

We accounted for five studies, presenting behavioral reactivity to 24 affective stimuli obtained from 931 participants with age ranging from 18 to 35 (*M* 22.37, *SD* 2.93). Of participants, 372 (40%) were female. The most frequently studied valence category was positive emotions (1676 reactions; 39.5%), then negative emotions (1444 reactions; 34.0%), and neutral conditions (1096 reactions; 25.80%). The regression models fit data well for approach/avoidance behavior, *PPp* = 0.480, 95% CI [− 6.368, 7.436], and for freezing/activation behavior *PPp* = 0.471, 95% CI [− 7.429, 7.167].

### Approach/avoidance behavior

**Figure 1:**
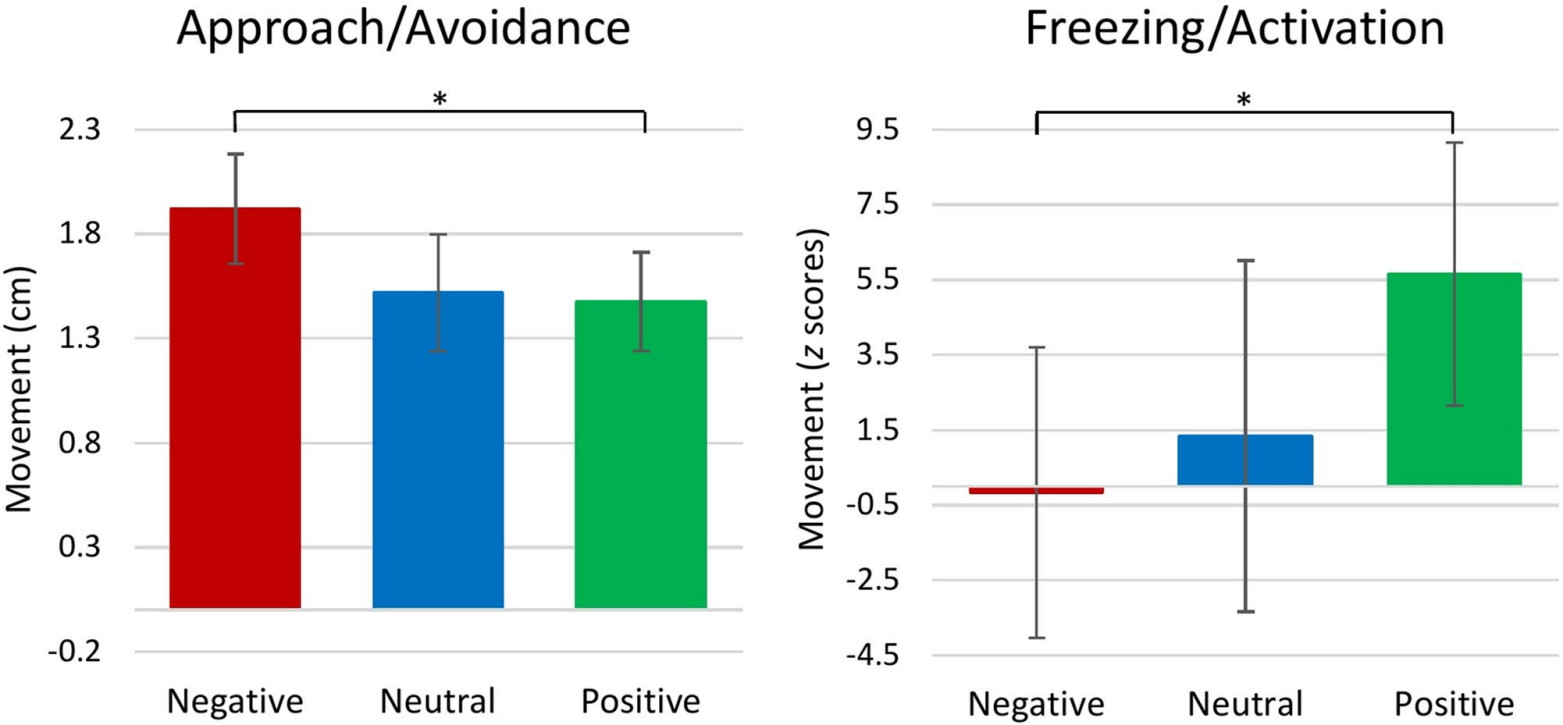
Emotional behavior reactivity for neutral, negative, and positive stimuli from baseline. Approach/Avoidance is expressed in centimeters as the change of the distance between head and camera; Freezing/Activation is expressed in *z* scores as the change of the total body sway; Error bars represent 95% confidence intervals of the means; Asterisks indicate significant differences between stimulus categories (*p < 0.05).

We found that the approach/avoidance behavior differs between negative and positive stimuli, Δβ = − 0.050, 95% CI [0.008, 0.093], *p* = 0.011. Individuals leaned backward more while watching negative stimuli (*M* − 1.92 cm, *SD* 4.06 cm) than while watching positive stimuli (*M* − 1.47 cm, *SD* 4.07 cm)(Fig. 1). However, approach/avoidance behavior to neutral stimuli (*M* − 1.52 cm, *SD* 4.10 cm) did not differ from the behavior to negative stimuli, β = − 0.045, 95% CI [− 0.091, 0.002], *p* = 0.030, and positive stimuli, β = 0.006, 95% CI [− 0.037, 0.051], *p* = 0.403.

### Behavioral freezing/activation

We found that the behavioral freezing/activation differed between negative and positive stimuli, Δβ = − 0.041, 95% CI [0.003, 0.079], *p* = 0.016. Individuals moved less while watching negative stimuli (*M* − 0.17, *SD* 67.65) than while watching positive stimuli (*M* 5.66, *SD* 66.15) (Fig. 1). However, behavioral freezing/activation to neutral stimuli (*M* 1.34, *SD* 72.56) did not differ from negative stimuli, β = − 0.010, 95% CI [− 0.050, 0.034], *p* = 0.327, and positive emotions β = 0.031, 95% CI [− 0.012, 0.071], *p* = 0.068.

## Discussion

We demonstrated how emotions impact the spontaneous head sway in seated individuals. We observed differences between responses to positive and negative stimuli. First, the head distance was shorter relative to positive stimuli than to negative stimuli. These findings are in line with the majority of previous studies^1,9,24^. It supports the biphasic approach to emotions, which suggests that emotions are organized around two motivational systems: approach oriented or appetitive and avoidance oriented or defensive systems^13^. Second, individuals moved less while facing negatively evaluated stimuli relative to positively evaluated stimuli. Again, this finding is consistent with the majority of the previous studies^9,16,21–23^. It supports that the emotional behavior is organized by its direction as well as intensity^13^. Thus, our findings provide evidence that corroborates previous research using relatively novel methods^9,16,21–24^. However, the observed effects should be interpreted as subtle as the effect sizes were small.

We did not find the differences between positive or negative stimuli relative to neutral stimuli. Some previous studies also showed no differences for approach/avoidance behavior and freezing/activation behavior^3,4,6,9,11,12,24^. This, again, indicates that it is easier to differentiate between extreme values than between extreme and intermediate values^23,24,26^.

Seeking to advance understanding of the emotional influence on complex human behavior, we used two novel methodological approaches. First, we used head movements as a reliable and ecologically valid index of the amount of spontaneous body sway during emotion elicitation in standing individuals^24^. We aimed to examine previous findings derived from stabilometric force platforms with using head movements as a commonly available, low-cost movement index that can be determined from the video recordings. We observed that the head measures were sensitive to experimental manipulation, as indicated by differences between positive and negative emotions. In Study 5, we included leg movements as an additional measure of behavioral freezing/activation. Due to our knowledge, it was the first attempt to account for lower limb spontaneous mobility as the measure of emotional behavior. While head movement might be restricted by chair headrest, armrest, or attached additional apparatus (e.g., electromyography or eye-tracking devices), the leg activity might be the easily available index of the behavioral reactivity.

Second, studies within the whole-body movement paradigm mainly reported data of participants standing on a force platform, whereas we focused on the body sway of seated participants. During quiet upright standing, the human body exhibits a small amount of spontaneous postural fluctuation, which can even be reduced in response to aversive stimuli. The reduction occurs because of contraction or stiffening of the muscles around the ankle joint^11,16,17^. We argue that the stiffening is not limited to ankle joints and would also occur in the upper body (e.g., contraction trapezius muscle)^36^, and should be visible in the seated postural responses. Studies accounting for muscle activity and spontaneous movements will be necessary to confirm this hypothesis. Since adults spend more than half of their day sedentary^28^, further studying seated postural responses would ecologically valid.

The study has several limitations. First, based on the previous study on standing individuals^24^, we assumed that seated individuals' video recordings would suffice to determine the influence of valence on emotional behavior. However, seated individuals are in a more restricted position than standing individuals and have fewer degrees of freedom in motion. Participants in our studies sat on the chair with a backrest that might limit their anterior–posterior body sway and an armrest that might limit their medial–lateral body sway. Thus, the subtle effect of emotions on spontaneous whole-body behavior might not be detectable while measured in a restricted body position. For instance, researchers found the effects of emotions on spontaneous behavior only in unipedal stance, but not bipedal stance, which indicates that the degrees of freedom of spontaneous motion matters^11^. This might explain why we found differences between the valence dimension's extremes—positive and negative emotions, but not from the neutral condition. More studies that compare whole-body standing movement and seated postural responses might support this hypothesis. Future studies may also use an experimental setup with the sitting ball without arm- and backrests to allow for additional movements while sitting. Second, we based our conclusions on data from video recordings instead of using the force platform that is the state-of-the-art method in behavioral literature. Future studies may validate the initial findings on the comparability of both methods^24^. Third, in our studies, we did not control for eye movements that would inform whether individuals focused on specific parts of the screen while watching the positive and negative stimuli. For instance, head movements might be stronger for stimuli where individuals had to switch their focus between different parts of the scene, i.e., to understand the social interaction between several individuals. In contrast, some negative stimuli might produce a stronger focus on one part of the screen (e.g., a dangerous animal). Thus, the complexity of the stimuli might interact with the effects of valence. Future studies might account for this limitation by controlling for eye movements or by selecting positive and negative stimuli that produce similar eye movements. Fourth, we compared positive, negative emotions, and neutral conditions, whereas the discrete emotion approach could yield more insight. Recent work has demonstrated that discrete emotions often differentially impact cognition and physiology even when of the same valence^35,37,38^. Furthermore, we used multiple film clips to elicit single emotion, which provides additional variation in the manipulation strength. However, this approach also generalizes the impact of emotional valence and limits the effect of using specific stimuli. Researchers might use our approach to re-examine findings from other laboratory experiments on discrete emotions, in which behavioral reactivity was not the main focus. Given that behavioral reactivity (with the exemption of facial expressions) gained relatively low popularity in affective science, reexaminations of existing data may result in an effective way to increase the number of examined emotion.

This study's strength is in the use of a novel method of movement analysis with a large number of participants. We evidenced that the analysis of head sway among participants in a seated position can reflect affective influences. As recording videos of sitting participants is affordable and easy, studies within the field of affective science might increasingly use this method to expand the scope of emotional behavior analysis.

## Methods

### Participants

Participants' characteristics are presented in Table 1. All studies were approved by and performed in accordance with guidelines and regulations of the Institutional Ethics Committee at Faculty of Psychology and Cognitive Science, Adam Mickiewicz University. Participants in our studies provided written informed consent and received cinema tickets for their involvement. The data for this investigation were collected from November 2016 to March 2019 in Poznan, Poland.

### Procedure

All five experiments began with a 5-min resting baseline. Participants were asked to wait for five minutes without doing any unnecessary actions. During the experiments, we elicited emotions (Table 1), presenting affective pictures or film clips. Unlike previous studies that used the head movement of standing individuals as an index of emotional behavior^24,26^, participants in our study sat on a chair with the back and armrests throughout all studies.

### Affective stimuli

#### Films

We used stimuli from the emotion-eliciting video clip databases to elicit emotions, with prior evidence of reliability and validity^39–42^. Each clip lasted two minutes. Within the sessions, clips were presented in a counterbalanced order. Table 1 presents which films were used to elicit emotions in the studies.

For positive emotions, we used validated movie clips: (1) *A fish called Wanda* (Unexpectedly, the owners of the house get into the house and discover Archie dancing naked); (2) *The visitors* (The visitors destroy the postman's car); (3) *When Harry met Sally* (Sally fakes an orgasm in the restaurant); (4) *The Dead Poets Society* (Students climb on their desks to manifest their solidarity with their teacher); (5) *Life is beautiful* (In a prisoner's camp, a father and a boy talk to the mother using a loudspeaker); (6) *Benny & Joone* (Benny plays the fool in a coffee shop); and (7) *Summer Olympic Games* (montage of moments showing athletes successful performance and their joyful reactions). We used films 1–6 in Study 1, films 1–3 in Study 2, and film 1 and 7 in Study 5.

For negative emotions, we used validated movie clips (1) *American History X* (A neo-nazi smashes a Black man's head on the curb and killing him); (2) *Man bites dog *(A hitman pulls out a gun, yelling at an old lady); (3) *In the name of the fathe*r (Scene of interrogation); (4) *The Blair Witch Project* (the characters die in an abandoned house); (5) *Seven* (the police find the decaying corpse); (6) *Dangerous minds* (the teacher tells the class that one of their classmates is dead); and (7) *The Champ* (boy cries at the death of his father). We used films 1–6 in Study 1, films 1–3 in Study 2, and film 1 & 7 in Study 5.

For neutral conditions, we used validated movie clips: (1) *Blue* 1 (A man clears out the drawers of his desk, or a woman walks in an alley); (2) *Blue* 2 (A woman goes up on an escalator, carrying a box); (3) *Blue* 3 (A person passes a piece of aluminum foil through the window of a car); (4) *Emperor* 1 (The Emperor talks with his teacher); (5) *Emperor* 2 (scenes from the city life); (6) *The lover* (the protagonist walks around the city); (7) *Twin Peaks: Fire Walk with Me* (the character sweeps the floor in the bar). We used films 1–5 & 7 in Study 1, films 1, 4 & 5 in Study 2, and film 2 in Study 5.

#### Pictures

In Study 3 and 4, we used validated pictures^31^ from the Nencki Affective Pictures System^43^. We selected three groups of items: high-approach positive affect (Faces340; Landscapes008, L023, L100, L110, L117, L,140, L149, Objects078, O081, O096, O183, O254, O291, O323, People108, P173, P189), low-approach positive affect (Animals099, A153, Faces076, F113, F179, F228, F232, F234, F238, F330, F332, F337, F344, F347, F353, F358, Objects192, O260), and neutral (Faces157, F166, F167, F309, F312, Landscapes012, L016, L024, L056, L061, L067, L076, L079, Objects112, O204, O210, O310, O314). In Study 3 & 4 we used the same set of pictures.

### Measures

#### Apparatus

**Figure 2:**
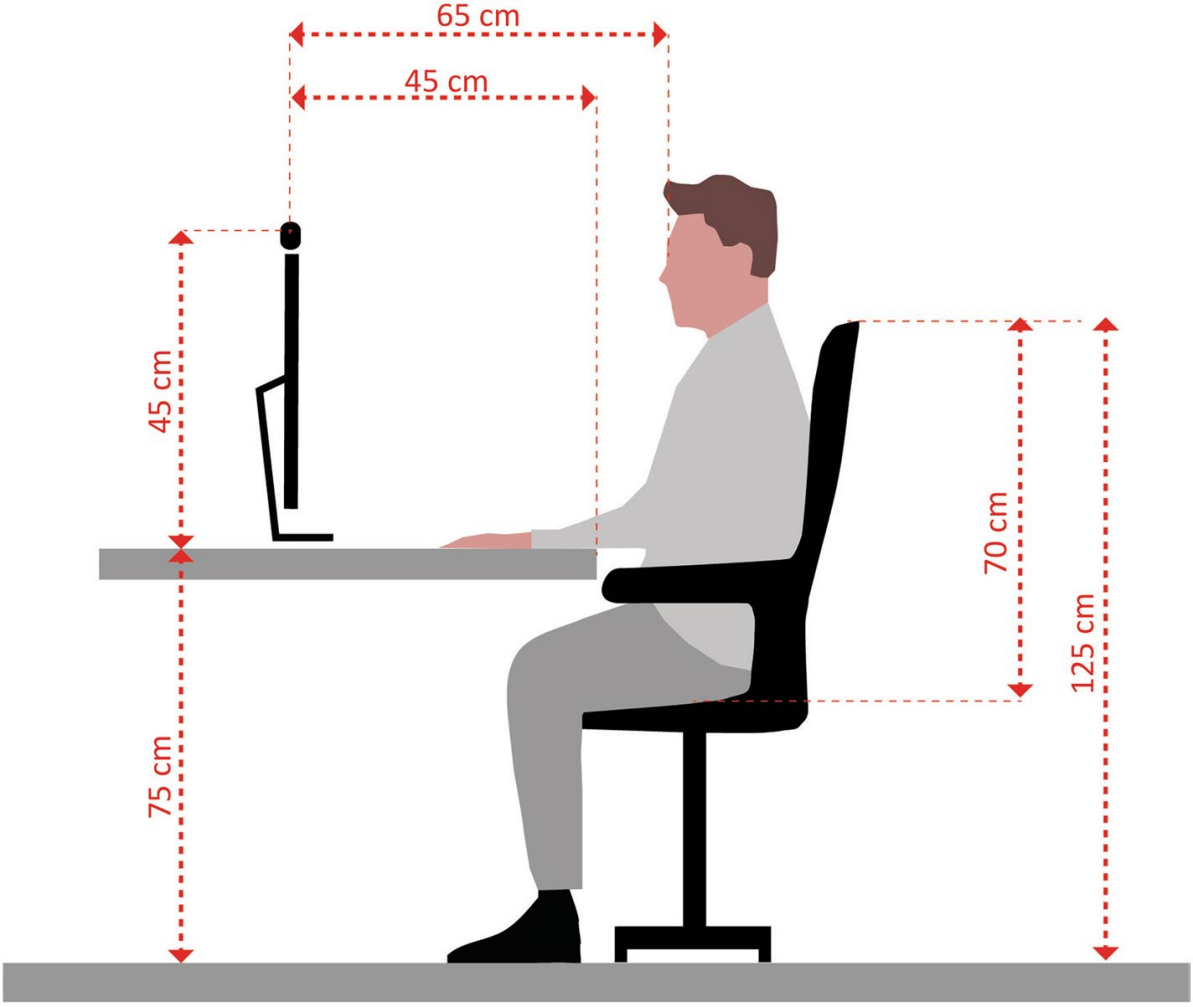
Schematic figure of the experimental setup. This figure was created by Katarzyna Janicka. The copyright of the figure is held by Katarzyna Janicka.

We recorded the head movements of each participant continuously using an HD camera mounted on the top of the PC screen. The distance from the camera to participants’ head was approximately 65 cm (Fig. 2). The video data were analyzed using facial expression analysis software Quantum Sense (Quantum CX, Poland)^44,45^, which uses computer vision algorithms to detect and determine the head positions in a three-dimensional space for each frame. Data was recorded with the sampling rate of 15 FPS in Study 1 and Study 2, 10 FPS in Study 3 and Study 4, and 24 FPS in Study 5. We also included leg movements in Study 5. We used four accelerometers (wGT3X-BT, Actigraph, USA) attached to left and right knee and left and right ankle to record physical activity continuously and unobtrusively.

#### Avoidance/approach behavior

We measured the head position along the anterior–posterior axis, as the distance of the head from the camera. We calculated the mean distance of the head from the camera from two moments in our studies: (1) the last two minutes of resting baseline to account for the part of the baseline that was the most proximal to the study, and (2) the last 2 min of emotion manipulation. To operationalize changes of avoidance/approach behavior, we used reactivity scores corrected for the baseline levels; thus, we subtracted the levels of the last 120 s of baseline from the level of the last 120 s of the emotion manipulation. We did this due to differential lengths of presentation of the pictures (3 min) and film clips (2 min) and to account for the part of manipulation that is usually more affected by emotions elicitation^40^. This is a validated approach in affective sciences in studies that compare responses to two different stimuli of different durations^46,47^. The head sway in the anterior direction (the positive difference between baseline and affective stimuli) indicates approach behavior, whereas the head sway in the posterior direction (the negative difference between baseline and affective stimuli) indicates avoidance behavior.

#### Behavioral freezing/activation

We measured behavioral freezing/activation with the total movement of head sway. First, we calculated the total movement of the head in *X, Y, Z* axis, based on the difference in head position in consecutive video frames. Second, we summed up the magnitude of head sway from two moments in our studies: last 2 min of baseline and last two minutes of emotion elicitation. To operationalize behavioral change of head sway to emotional stimuli, we used reactivity scores corrected for baseline levels. The decreased total body sway (the negative difference between baseline and affective stimuli) indicates behavioral freezing, whereas the increased total head sway (the positive difference between baseline and affective stimuli) indicates behavioral activation. In Study 5, we also calculated the magnitude of legs movement as the sum of all four joints sways in *X, Y, Z*-axis. The internal reliability for four joints movements was satisfactory, α = 0.84. To test the impact of emotions on behavioral freezing/activation collected from two movement sources (head and legs), we converted the data from each source of behavioral activity into the *z*-scores, and we merged them into a single variable.

### Analytical strategy

To summarize data from our multiple studies showing how emotions influence behavioral reactivity, we used three-level regression models using mPlus 8.0^48^. We ran the three-level regression models to account for dependency within-person (level 2) and within a study (level 3). Furthermore, we used three-level regression models to determine global trends across studies to account for dependency between the studies (e.g., studies conducted by the same laboratory, by the same team of researchers). We regressed the approach/avoidance behavior and freezing/activation behavior on the experimental condition (positive, negative, and neutral stimuli). We dummy-coded the experimental conditions such that significant differences in the model accounted for differences relative to the neutral condition. Furthermore, the behavioral reactivity to emotion category (positive emotions vs. negative emotions) was considered as significantly different if the 95% confidence intervals of the difference between regression coefficients in subgroups did not include zero. We ran the analysis with the Bayesian correction estimator (Bayes), and we used the Bayesian Posterior Predictive (*PPp*) to evaluate model fit. A well-fitting model should have a *PPp* value around 0.50 in combination with symmetric 95% credibility intervals centering on zero^49,50^. We removed outliers above z-scores higher than 3.29^51^, resulting in 721 data points (4.90%) removed from the initial data sample.

## Supplementary Information

Supplementary Information.

## Data Availability

The datasets generated during the current study are available from the corresponding author upon request. All data analyzed during this study are included in this published article (Supplementary Information file).
