# Supplementary material for: Head movement differs for positive and negative emotions in video recordings of sitting individuals
Source: Sci Rep. 2021 Apr 1;11:7405. doi: 10.1038/s41598-021-86841-8 (PMC8016997; doi:10.1038/s41598-021-86841-8)
Supplement: Supplementary file 1 — Supplementary Information. [file 41598_2021_86841_MOESM1_ESM.zip › EmotionBehviorSupp/Title page.docx]

**Head movement differs for positive and negative emotions in video recordings of sitting individuals**

**Maciej Behnke^1,*^, Nadia Bianchi-Berthouze^2^, Lukasz D. Kaczmarek^1^**

^1^Adam Mickiewicz University, Faculty of Psychology and Cognitive Science, Poznan, 61-664, Poland

^2^University Collage London, Interaction Centre, London, WC1E 6EA, United Kingdom

^*^macbeh@amu.edu.pl
